# CDA gene silencing regulated the proliferation and apoptosis of chronic myeloid leukemia K562 cells

**DOI:** 10.1186/s12935-018-0587-y

**Published:** 2018-07-09

**Authors:** Xiao-Fang Wei, You-Fan Feng, Qiao-Lin Chen, Qi-Ke Zhang

**Affiliations:** Department of Hematology, Gansu Provincial Hospital, No. 204, Donggang West Road, Lanzhou, 730000 Gansu People’s Republic of China

**Keywords:** Cytidine deaminase, Chronic myeloid leukemia, K562 cells, Proliferation, Apoptosis, Gene silencing

## Abstract

**Background:**

As a disease of hematopoietic stem cell, chronic myeloid leukemia (CML) possesses unique biological and clinical features. However, the biologic mechanism underlying its development remains poorly understood. Thus, the objective of the present study is to discuss the effect of cytidine deaminase (CDA) gene silencing on the apoptosis and proliferation of CML K562 cells.

**Methods:**

CDA mRNA expression was detected by reverse transcription-quantitative polymerase chain reaction (RT-qPCR), and enzymatic activity of CDA was measured by a nuclide liquid scintillation method. RT-qPCR and Western blot analysis were used to detect CDA mRNA and protein expression. Cell proliferation, apoptosis and cell cycle were measured by CCK-8 assay and flow cytometry. The expression of proteins relevant to cell proliferation, apoptosis and cell cycle was measured by Western blot analysis. Tumor xenografts were implanted in nude mice to verify the effect of CDA silencing on tumor growth in vivo.

**Results:**

CML and AL patients showed increased mRNA expression and enzymatic activity of CDA. Compared with the blank group, the mRNA and protein expression of CDA in the shRNA-1 and shRNA-2 groups decreased significantly. As a result, the proliferation of K562 cells was inhibited after CDA silencing and the cells were mainly arrested in S and G2 phases, while the apoptosis rate of these cells was increased. In addition, CDA gene silencing in K562 cells led to down-regulated p-ERK1/2, t-AKT, p-AKT and BCL-2 expression and up-regulated expression of P21, Bax, cleaved caspase-3/total caspase-3 and cleaved PARP/total PARP. Finally, CDA gene silencing inhibited tumor growth.

**Conclusion:**

Our study demonstrated that CDA gene silencing could inhibit CML cell proliferation and induce cell apoptosis. Therefore, CDA gene silencing may become an effective target for the treatment of leukemia.

## Background

Chronic myeloid leukemia (CML) is a hematopoietic malignant disease characterized by the presence of the Philadelphia chromosome (Ph) due to the reciprocal translocation of chromosomes 9 and 22, resulting in the formation of a BCR–ABL fusion onco-protein [1–3]. As a myelo-proliferative neoplasm, the incidence of CML is higher than 2/100,000 [4]. In addition, the incidence of CML in females is lower than that in males [5]. The BCR–ABL fusion protein leads to the pathogenicity of CML, while the therapies targeting the BCR–ABL protein have encountered many difficulties [6]. Besides, based on the characteristics related to CML, the prognostic scoring systems of CML tend to classify patients into different risk groups [7]. Currently, the tyrosine kinase inhibitor treatment has been regarded as the fundamental therapy for CML, while imatinib and dasatinib or nilotinib have been used in first-line and second-line treatments, respectively [8]. In addition, gene therapy has become more common recently in the treatment of CML. For example, RNA interference or gene silencing has been used as new strategies for CML treatment [9].

Cytidine deaminase (CDA) is a leading enzyme that participates in the metabolism of gemcitabine, a pyrimidine analog used for chemotherapy in treating tumors such as biliary tract cancer [10, 11]. According to the crystal structures and possible amino acid sequences of CDA in different species, CDA has been divided into two types, i.e., a homodimeric class and a homotetrameric class [12]. CDA expression is used to classify new subclass of cancers. For anti-tumor drugs, loss of CDA expression seems to act as a new predictive marker of cancer susceptibility [13]. In terms of RNAi-mediated gene silencing, undergoing clinical trials have tried to use it as an alternative to traditional chemotherapies in cancer treatment [14]. In a related study, cytarabine was found to have high efficacy in the treatment of acute myeloid leukemia and its efficacy was delaminated by CDA [15]. Nevertheless, the underlying mechanism of CDA gene silencing in the biological processes of K562 cells remains to be clarified. Therefore, in this study, we aimed to investigate the effects of CDA gene silencing of the apoptosis and proliferation of CML K562 cells.

## Materials and methods

### Study subjects

Between January 2012 and January 2015, all leukemia patients diagnosed in the hematology department of Gansu Provincial Hospital were screened. Eventually, 112 CML patients were enrolled in this study, including 70 males and 42 females with a mean age of 34 years (range from 18 to 47 years). This study also enrolled 106 acute leukemia (AL) patients, including 62 males and 44 females with a mean age of 32 years (range from 19 to 48 years), as well as 101 donors for hematopoietic stem cell transplantation, including 53 males and 48 females with a mean age of 32 years (range from 18 to 43 years). All leukemia patients were diagnosed based on MICM (morphology, immunology, cytogenetics, molecular biology and biochemistry) [16]. Bone marrow mononuclear cells were separated using a lymphocyte separation medium (Ficoll separating liquid) (Thermo Fisher Scientific, San Jose, California, USA), while collected cell clusters were used immediately or preserved at − 80 °C for future use. This study was approved by the ethics committee of Gansu Provincial Hospital, and all patients had signed informed consent.

### Reveres transcription-quantitative polymerase chain reaction (RT-qPCR)

The total RNA in collected cell samples was extracted using a Trizol-based one-step method according to the instructions of Trizol reagent (Invitrogen Inc., Carlsbad, CA, USA). RNA was dissolved in ultra-pure water pre-treated by diethylpyrocarbonate (DECP). RNA quality was evaluated by measuring its absorbance at 260 and 280 nm on an ND-1000 ultraviolet/visible spectroscopy (UV/VIS) spectrophotometer (NanoDrop Technologies, Wilmington, DE, USA). Subsequently, the concentration of RNA was adjusted for RT-qPCR. The extracted RNA was reversely transcribed using a two-step method according to the instructions of a reagent kit (Fermentas Inc., Hanover, MD, USA). The reaction conditions of RT were as follows: 70 °C for 10 min, ice bathed for 2 min, 42 °C for 60 min, and 70 °C for 10 min. The obtained cDNA was stored in a − 80 °C freezer. The TaqMan probe method was used in qPCR, and the reaction system was prepared according to the instructions of a reagent kit (Fermentas Inc., Hanover, MD, USA). The upstream and downstream sequences of CDA primers were 5′-CCGTCTCAGAAGGGTACAAG-3′ and 5′-GACAATATACGTACCATCCGG-3′. The reaction conditions of qPCR were as follows: pre-denaturation at 95 °C for 30 s, and 40 cycles of denaturation at 95 °C for 10 s, annealing at 60 °C for 20 s, and extension at 70 °C for 10 s. An RT-qPCR instrument (American Bio-Rad company, model, is Bio-Rad iQ5) was used to carry out RT-qPCR, while glyceraldehyde-3-phosphate dehydrogenase (GAPDH, upstream sequence: 5′-ATTCAACGGCACAGTCAAGG-3′, downstream sequence: 5′-GCAGAAGGGGCGGAGATGA-3′) was used as an internal reference. The relative expression of CDA was calculated using 2^−∆∆Ct^, and each experiment was repeated 3 times.

### Nuclide liquid scintillation

After thawing, the sample cells were rinsed twice with phosphate buffered saline (PBS). Subsequently, the cells (1 × 10^7^) were placed into frozen tubes, followed by the addition of 1 ml CDA buffer solution (pH 7.50, 50 mmol/l Tris–HCl, 2 mmol/l DTT, and 10% glycerol) (Beijing Solarbio Science & Technology Co., Ltd). The cell membranes were ruptured after three freeze–thaw cycles in liquid nitrogen, after which the cells were centrifuged at 1500*g* and 4 °C for 10 min. Subsequently, 0.1 ml 3*H*-cytidine (0.03 μ Ci) (Hoffmann-La Roche Ltd., Basel, Switzerland) was added into 0.5 ml supernatant and incubated at 37 °C for 2 h. In the next step, the mixture was dripped slowly into a resin column and slowly chased with 20 ml distilled water. Afterwards, 3*H*-uridine was collected into a disinfected tube and mixed with 3 ml liquid scintillation cocktail. The value of corrected counts per minute (CCPM) was then measured on a liquid scintillation counter.

### Cell culture

CML K526 cells were purchased from Shanghai cell bank of the Chinese Academy of Sciences and maintained in a Roswell Park Memorial Institute (RPMI)-1640 (Gibco Company Grand Island, NY, USA) supplemented with 10% fetal bovine serum (FBS) (Hyclone Company, Logan, UT, USA). During this study, the cells were cultured in a 5% CO_2_ incubator (Thermo Fisher Scientific, San Jose, California, USA. model, Thermo Scientific 8000) at 37 °C and 95% humidity. During cell passage, the original medium was removed through centrifugation at 1000 rpm and the cells were rinsed twice with PBS before adding a new RPMI-1640 medium (containing 10% FBS).

### Construction of CDA gene silencing and CDA overexpression models

Based on the CDA gene sequence in GenBank, two segments of CDA specific target sequences were selected using an RNAi design tool on http://www.oligotide.com and short hairpin RNAs (shRNAs) targeting these sequences (shRNA-1 and shRNA-2) were synthesized in conjunction with a negative control sequence (shRNA-con) (Table 1) by Shanghai Sangon Biological Engineering Technology & Services Co., Ltd. (Shanghai, China). As confirmed by homology sequence inquiry through basic local alignment search tool (BLAST), the well-designed target sequence of shRNA for CDA gene showed no homology with other sequences. The reversed complementary sequence of this target sequence was synthesized, and the transcription of antisense strand was terminated using transcriptional termination TTTTTT of RNA polymerase III. The above RNA was inserted to a pBS/U6-Neo plasmid and transformed into competent DH5 alpha bacteria, after which anti-neomycin positive clones were selected for plasmid amplification. The correctness of the constructed plasmids was checked by double-enzyme digestion of *Sal*I and *Hin*dIII. A search of CDA gene sequence was conducted using National Center of Biotechnology Information (NCBI), and the result showed that its serial number was NM_001785, 441 bp in the coding sequence region. The sequence of CDA gene was retrieved from cells, and a CDA overexpression vector was constructed. After identification with double enzyme digestion, DNA sequencing analysis was performed to confirm that the correct clone was cultured before the plasmid was extracted for cell transfection.

**Table 1:** Interfering RNA (siRNA) sequences for CDA gene

Groups	Sequences
shRNA-1	5′-TGAGAGAGTTTGGCACCAA-3′
shRNA-2	5′-CAGTGACATGCAAGATGAT-3′
shRNA-con	5′-GTTCTCCGAACGTGTCACG-3′

*CDA* cytidine deaminase

### Cell transfection and grouping

The cells were assigned into the following groups: a blank group, a shRNA-con group, a shRNA-1 group, a shRNA-2 group, and a group of over-expressed CDA. K526 cells in the logarithmic growth phase were selected for transfection. During transfection, shRNA plasmids (at a final concentration of 50 nM) were diluted in 250 µl serum-free Opti-MEM medium (Gibco Company, Grand Island, NY, USA), gently mixed and incubated at room temperature for 5 min. At the same time, 5 µl Lipofectamine 2000 were diluted in 250 µl serum-free Opti-MEM medium, gently mixed and incubated at room temperature for 5 min. The above two solutions were then mixed, incubated at room temperature for 20 min and added onto the cells. After 24–48 h transfection, the cells were collected for further experiments.

### Cell counting kit-8 (CCK-8) assay

After 24 h of transfection, cells were centrifuged to remove the original medium, rinsed twice with PBS and made into a single cell suspension. After counting, the cells were seeded into a 96-well plate in 100 μl/well medium at a density of 3–6 × 10^3^ cells per well. Each cell treatment was tested in triplicate. During the assay, each well was added with 10 µl of CCK-8 solution and cultured for 4–6 h (American Sigma Company). The optical density (OD) of each well was detected at 450 nm using an enzyme-linked immuno-sorbent assay (ELISA) after 24, 48 and 72 h of incubation. Cell viability curves were drawn using time as the abscissa and survival rate (%) as the ordinate.

### Clonogenic assay

The cells were detached with trypsin, suspended and counted. Afterwards, the cells were seeded into a 6-well plate at a density of 1000 cells/well, and cultured in a semi-fixed medium under 5% CO_2_ and 37 °C. After 2 weeks, the cells were stained with crystal violet, and the number and size of cell colonies were observed. The experiment was repeated 3 times.

### Flow cytometry

Detection of cell cycle: after 48 h of transfection, the cells were collected, rinsed 3 times with ice-cold PBS and centrifuged to remove the supernatant. The concentration of the cells was adjusted to approximately 1 × 10^5^/ml. Subsequently, the cells were fixed in 1 ml ice-cold 75% ethanol at 4 °C overnight. Before staining, the cells were rinsed twice with PBS, added into 100 μl RNaseA and incubated at 37 °C for 30 min in the dark. Subsequently, the cells were stained with 400 μl PI (Sigma-Aldrich Chemical Company, St Louis MO, USA) at 4 °C for 30 min in the dark. Cell cycle was detected by flow cytometry (American BD Biosciences Company. Model, FACSCanto II) at 488 nm.

Detection of cell apoptosis: after 48 h of transfection, the cells were collected into flow tubes and centrifuged at 178 g for 5 min. Subsequently, the cells were rinsed 3 times with ice-cold PBS and centrifuged again. In accordance with the instruction of an Annexin-V-FITC apoptosis determination kit (Sigma-Aldrich Chemical Company, St Louis MO, USA), 150 μl binding buffer and 5 μl Annexin-V-FITC were added into each tube. After oscillation, the cells were incubated in the dark at room temperature for 15 min. Subsequently, another 100 μl of binding buffer and 5 μl PI stain (Sigma-Aldrich Chemical Company, St Louis MO, USA) were added into each tube. After oscillation, cell apoptosis was detected by flow cytometry.

### Western blot analysis

Cells were collected and incubated with a 1 × SDS lysis buffer (Beyotime Biotechnology Co., Shanghai, China). The extracted protein was heated at 100 °C for 5 min and the samples (20 μl) were loaded onto 10% polyacrylamide gel electrophoresis (PAGE). Subsequently, the protein was transferred at voltage of 48 V onto a polyvinylidene fluoride (PVDF) membrane for 3.5 h, followed by incubation with 5% bovine serum albumin (BSA) at room temperature for 2 h to block the membrane. After being rinsed by 1× tris-buffered saline with tween 20 (TBST), the membrane was incubated at 4 °C overnight with rabbit anti-human primary antibodies [CDA, t-AKT, t-extracellular signal-regulated kinase (ERK)1/2, p-ERK1/2, phosphatidylinositol-3 kinase (PI3K), B cell lymphoma/leukemia-2 (BCL-2), BCL-XL, bcl-2-associated X (Bax), p21, cleaved caspase-3, total caspase-3, cleaved poly (ADP-ribose) polymerase (PARP), and total PARP, all diluted by 1: 500, (Cell Signaling Technologies (CST), Beverly, MA, USA)]. Glyceraldehyde-3-phosphate dehydrogenase (GAPDH) antibody was used as an internal reference. After washing with 1 × TBST, the cells were incubated at room temperature for 1 h with peroxidase labeled goat anti-rabbit secondary antibody (1:2000) (Santa Cruz Biotechnology, Inc, Santa Cruz, CA, USA). After washing the cells with TBST again, chemiluminescence reagents (Thermo Fisher Scientific, California, USA) were added onto the cells and incubated for 1 min. The enhanced chemiluminescence (ECL) of all protein bands was visualized and recorded for subsequent Western blot analysis.

### Tumor xenograft implantation in nude mice

Specific pathogen free (SPF) nude mice (body weight: 18–25 g; age: 5–6 weeks old) purchased from Hunan SLAC Jingda Laboratory Animal Co., Ltd. (Changsha, Hunan, China) were raised in a key laboratory for SPF animals. The mice were housed in a SPF grade chamber under aseptic laminar flow and a constant temperature (22–25 °C)/humidity (55 ± 5%). All food, water and padding for the mice were sterilized. In this study, 9 nude mice were allocated into a blank, a shRNA-con and a shRNA-1 group, with 3 mice in each group. Before tumor inoculation, the back of each nude mouse was cleaned with 75% alcohol, and 0.2 ml of cell suspension (10^6^ cells/ml) were injected into each mouse using a skin test needle. The maximum diameter (a) and the maximum transverse diameter (b) of the tumor were measured every 3 days after inoculation, and the volume of the tumor (V = ab^2^/2) was calculated. The diet, body shape and defecation of nude mice were observed. After 28 days of inoculation, all nude mice were sacrificed by cervical dislocation. Subsequently, the tumor was isolated and weighed to calculate the tumor volume.

### Statistical analysis

SPSS 21.0 software (IBM Corp., Armonk, NY, USA) was used for data analysis. The measurement data were expressed as mean ± standard deviation. Comparison between two groups was conducted by *t* test. One-way analysis of variance (ANOVA) was used for comparing multiple groups. *p* < 0.05 was considered statistically significant.

## Results

### CDA mRNA and protein expression was increased in CML and AL patients

**Fig. 1:**
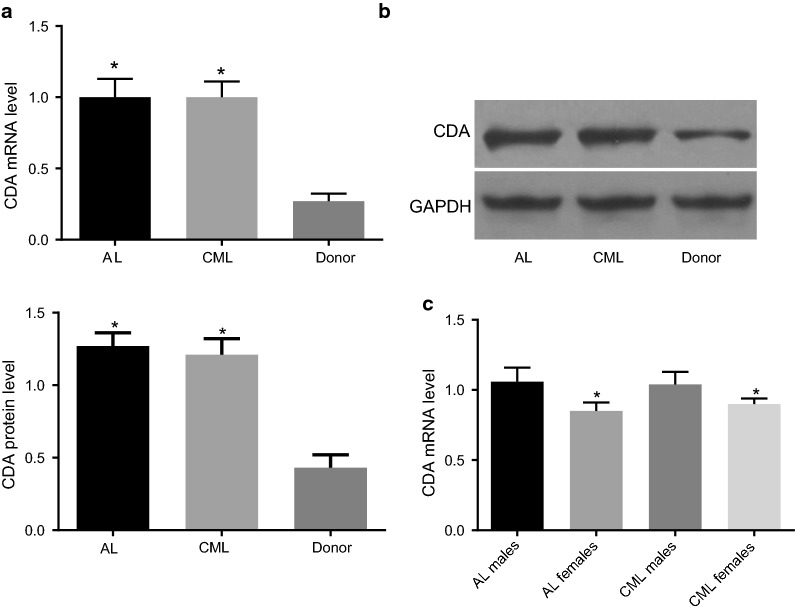
Higher mRNA expression of CDA was found in leukemia patients compared with that in the donors for hematopoietic stem cell transplantation. **a** CDA mRNA levels in leukemia patients and donors for hematopoietic stem cell transplantation (CDA mRNA level in leukemia patients was higher than that in the donors for hematopoietic stem cell transplantation); **b** CDA protein level in leukemia patients and donors for hematopoietic stem cell transplantation (CDA protein level in leukemia patients was higher than that in the donors for hematopoietic stem cell transplantation); **c** differential expression of CDA gene in AL patients and CML patients of different genders; the data were expressed as mean ± standard deviation, n = 3, and One-Way ANOVA was used to analyzed the data. *Compared to the donors for hematopoietic stem cell transplantation, *p* < 0.05; ^#^compared to male patients, *p* < 0.05. *CDA* cytidine deaminase, *CML* chronic myeloid leukemia, *AL* acute leukemia

RT-qPCR was used to detect CDA mRNA expression in bone marrow mononuclear cells of 112 CML and 106 AL patients, as well as in 101 donors of hematopoietic stem cell transplantation. The results indicated that, compared with donors of hematopoietic stem cell transplantation, CML and AL patients had increased CDA mRNA expression (all *p* < 0.001), which was about 2.70 and 2.69 times that in the donors of hematopoietic stem cell transplantation, respectively. CDA mRNA expression between CML and AL patients showed no significant difference (Fig. 1). The results of Western blot analysis showed that, compared with donors of hematopoietic stem cell transplantation, CML and AL patients had increased CDA protein expression (*p* < 0.001), which was about 1.95 and 1.32 times that in donors of hematopoietic stem cell transplantation, respectively. In addition, there was no difference between CML and AL patients in terms of CDA mRNA expression. Among 106 AL patients, there were 74 males and 32 females. The expression of CDA gene in male AL patients was significantly higher than that in female patients (*p* < 0.001). Among 112 CML patients, there were 76 males and 36 females. The expression of CDA gene in male CML patients was significantly higher than that in female patients (*p* < 0.001).

### Enzymatic activity of CDA was increased in CML and AL patients

Enzymatic activity of CDA was measured by the nuclide liquid scintillation method. As shown in Table 2 and compared with donors of hematopoietic stem cell transplantation, CML and AL patients showed an increased enzymatic activity of CDA (all *p* < 0.001), while the enzymatic activity of CDA between CML and AL patients showed no significant difference. The enzymatic activity of CDA in male AL and CML patients was significantly higher than that in female patients (*p* < 0.001, *p* < 0.001).

**Table 2:** Comparison of the enzymatic activity of CDA

Providers	Number	CDA (CCPM)
AL	106	1375.76 ± 321.69*
CML	112	1452.52 ± 432.43*
Donors	101	923.42 ± 269.02
AL males	74	1542.52 ± 202.65
AL females	32	990.97 ± 186.72^#^
CML males	76	1666.58 ± 344.66
CML females	36	1000.62 ± 170.17^#^

The data were expressed as mean ± standard deviation, n = 3, and One-Way ANOVA was used to analyzed data; CDA, Cytidine deaminase

*AL* acute leukemia, *CML* chronic myeloid leukemia

* Compared with the hematopoietic stem cell transplant donors, *p* < 0.05

^#^Compared to male patients, *p* < 0.05

### CDA gene silencing decreased CDA mRNA and protein expression

**Fig. 2:**
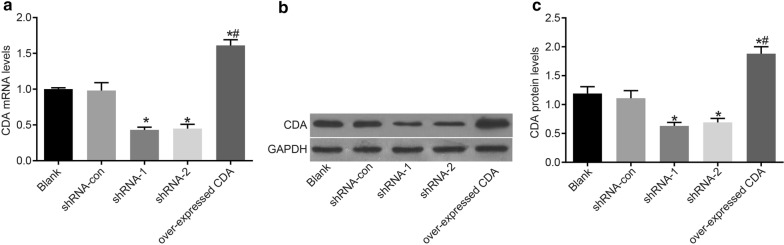
mRNA and protein expression of CDA was decreased in K562 cells after transfection with CDA shRNA. **a** CDA mRNA expression in each group; **b** gray value of CDA protein band; **c** CDA protein expression in each group; the data were expressed as mean ± standard deviation, n = 3, and One-Way ANOVA was used to analyzed the data. *Compared with the blank group, *p* < 0.05; ^#^compared with the shRNA-1 group, *p* < 0.05. *CDA* cytidine deaminase

RT-qPCR and Western blot analysis were employed to measure CDA mRNA and protein expression in post-transfection cells. As shown in Fig. 2 and compared with the blank group, CDA mRNA and protein expression was increased significantly (*p* = 0.0031) in the group of over-expressed CDA, with CDA mRNA and protein expression increased by 0.63- and 0.58-fold, respectively. In addition, CDA mRNA and protein expression in the shRNA-con group showed no significant change, while CDA mRNA and protein expression significantly decreased in the shRNA-1 and shRNA-2 groups (*p* < 0.001, *p* < 0.001). Overall, the mRNA expression of CDA decreased by 0.56- and 0.54-fold in shRNA-1 and shRNA-2 groups, respectively, while the protein level of CDA decreased by 0.47- and 0.42-fold, respectively. No significant difference was observed between shRNA-1 and shRNA-2 groups in terms of CDA mRNA and protein expression. The levels of CDA mRNA and protein in the group of over-expressed CDA were significantly higher than those in the shRNA-1 group (*p* < 0.0001). Therefore, the transfection of CDA-shRNA decreased CDA mRNA and protein expression.

### CDA gene silencing inhibited cell proliferation

**Fig. 3:**
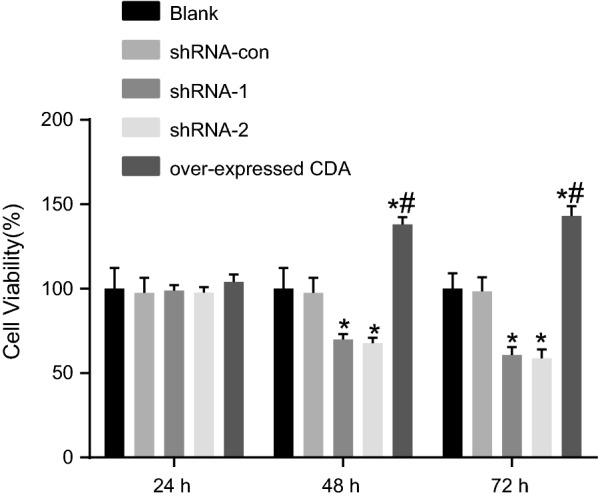
CDA shRNA inhibited cell proliferation. The data were expressed as mean ± standard deviation, n = 3, and One-Way ANOVA was used to analyzed the data. *Compared to the blank group, *p* < 0.05; ^#^compared with the shRNA-1 group, *p* < 0.05. *CDA* cytidine deaminase

Cell proliferation was measured by CCK-8 and the results are shown in Fig. 3. There was no statistical difference in OD, which reflected the level of cell proliferation, among different groups at 24 h. The OD value between the blank and shRNA-con groups showed no statistical difference at any time. Compared with the blank group, cell proliferation was remarkably inhibited in the shRNA-1 and shRNA-2 groups at 48 h (all *p* < 0.05), accompanied by declined survival rates of (30.18 ± 11.28)% and (32.64 ± 12.83)%, respectively. Cell proliferation was remarkably promoted in the group of over-expressed CDA, with its survival rate increased to (38.12 ± 11.78)%. At 72 h, cell proliferation was still inhibited in the shRNA-1 and shRNA-2 groups (all *p* < 0.05), with survival rates declined to (39.27 ± 11.40)% and (41.17 ± 13.54)%, respectively. Cell proliferation was remarkably promoted in the group of over-expressed CDA, with its survival rate increased to (43.16 ± 6.04)%. The cell growth in the group of over-expressed CDA was significantly faster than that in the shRNA-1 group and the difference was statistically significant (all *p* < 0.05). The cell proliferation between the shRNA-1 and shRNA-2 groups showed no significant difference at three time points (all *p* > 0.05). Therefore, cell proliferation was repressed by CDA gene silencing.

### Decreased clone cells in the shRNA-1 and shRNA-2 groups

**Fig. 4:**
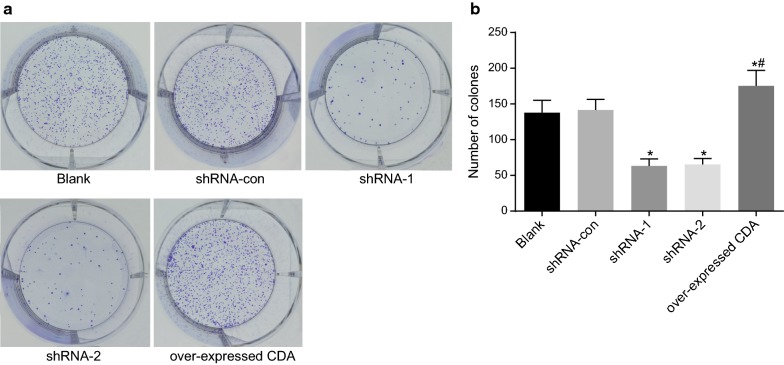
CDA gene silencing inhibited the formation of clone cells. **a** Clone cells in each group; **b** histogram of clone cells in each group; the data were expressed as mean ± standard deviation, n = 3, and One-Way ANOVA was used to analyzed the data. *Compared to the blank group, *p* < 0.05; ^#^compared with the shRNA-1 group, *p* < 0.05. *CDA* cytidine deaminase

Clone cells were assessed sing the Clonogenic assay (Fig. 4). Compared with the blank group, the number of clone cells in the group of over-expressed CDA was significantly increased. The shRNA-con group showed no significant change, while the number of clone cells decreased remarkably in the shRNA-1 and shRNA-2 groups (*p *< 0.001, *p *< 0.001). No significant difference was observed between the shRNA-1 and shRNA-2 groups. The number of clone cells in the group of over-expressed CDA was significantly higher than that in the shRNA-1 group (*p *< 0.0001). Therefore, CDA gene silencing reduced the number of clone cells.

### More cells were arrested in the S and G2 phases after CDA gene silencing

**Fig. 5:**
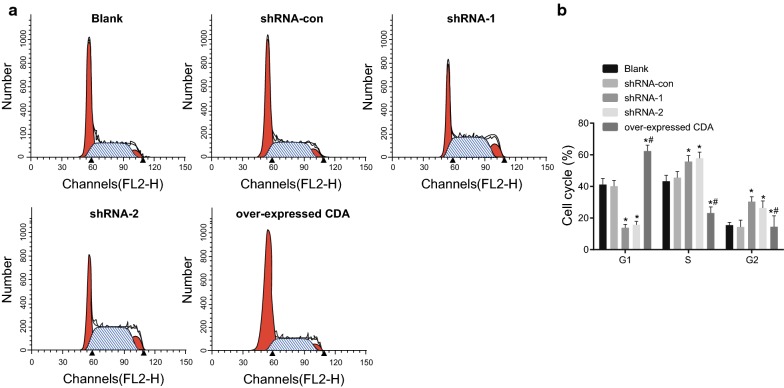
More cells were arrested in the S and G2 phases after transfection with CDA shRNA. The shRNA-1 and shRNA-2 groups exhibited a decreased number of cells in the G1 phase, but increased number of cells in the S and G2 phases. **a** Cell cycle in each group; the red region refers to the G1/G2 phase, and the gray region refers to the S phase; **b** histogram analysis of cell cycle distribution in each group; the data were expressed as mean ± standard deviation, n = 3, and One-Way ANOVA was used to analyzed the data. *Compared with the black group, *p* < 0.05. *CDA* cytidine deaminase

Flow cytometry was adopted for the detection of cell cycle. As shown in Fig. 5, flow cytometry showed that the proportions of cells in the G1 phase among the blank, shRNA-con, shRNA-1, shRNA-2 and group of over-expressed CDA were (41.19 ± 3.82)%, (40.08 ± 3.74)%, (13.90 ± 2.12)%, (15.75 ± 2.15)% and (62.36 ± 3.74)%, respectively. For these groups, the proportions of cells in the S phase were (43.32 ± 3.73)%, (45.56 ± 3.87)%, (55.76 ± 3.82)%, (57.85 ± 3.85)%, and (23.14 ± 3.87)%, respectively, and the proportions of cells in the G2/M phase were (15.49 ± 1.83)%, (14.36 ± 4.33)%, (30.34 ± 3.17)%, (26.40 ± 4.46)% and (14.50 ± 6.87)%, respectively. Compared with the blank group, the proportion of G1 cells increased significantly (*p *< 0.001) in the group of over-expressed CDA, whose proportion of cells in the S (*p *< 0.001) and G2 phases decreased significantly (*p *< 0.001). The shRNA-1 and shRNA-2 groups showed a decreased number of cells in the G1 phases (*p *< 0.001, *p *< 0.001), but an increased number of cells in S (*p *< 0.001, *p *< 0.001) and G2 phase (*p *< 0.001, *p *< 0.001). No obvious difference was observed in the shRNA-con group. The difference of cell cycle distribution between the shRNA-1 and shRNA-2 groups showed no statistical significance. Therefore, CDA gene silencing blocked cells in S and G2 phases.

### CDA gene silencing induced cell apoptosis

**Fig. 6:**
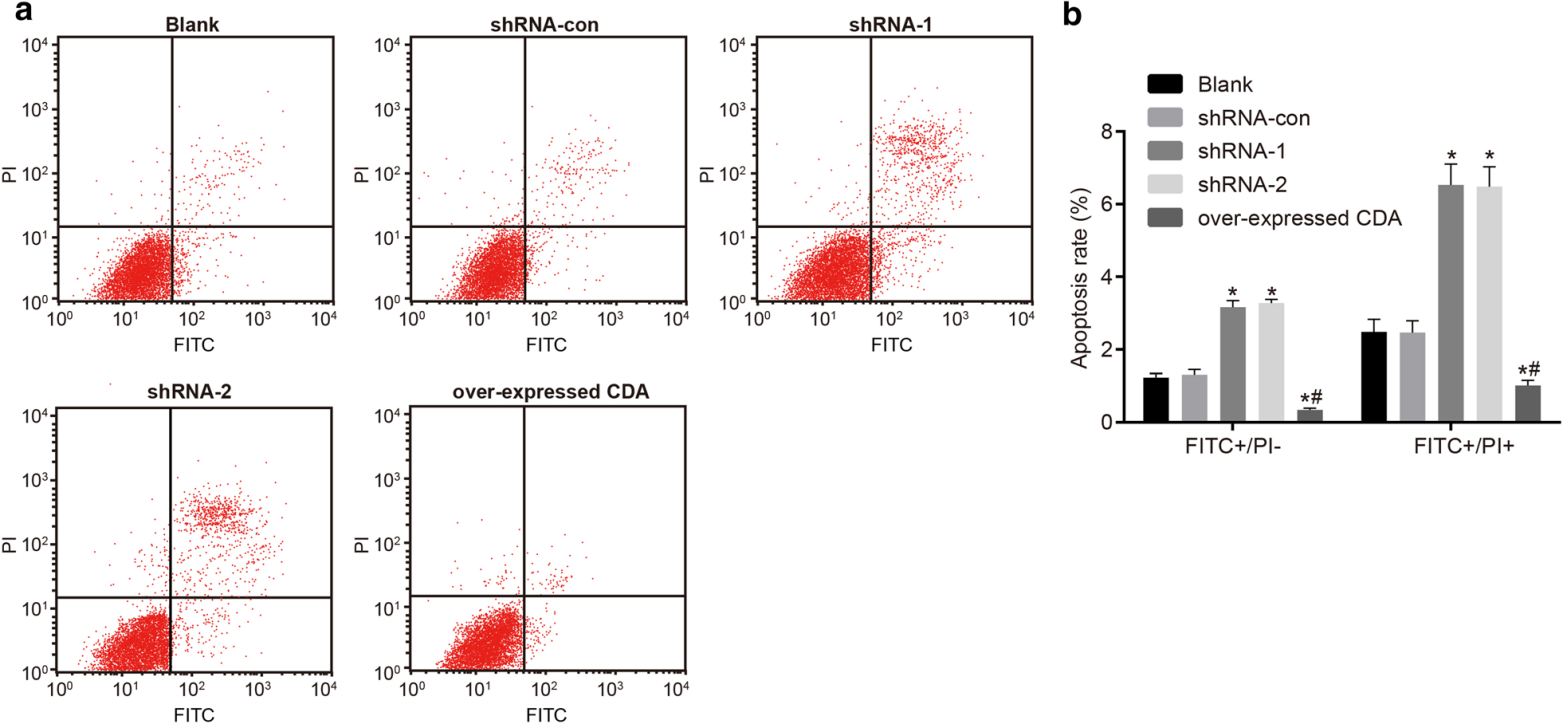
Cell apoptosis was promoted after transfection with CDA shRNA. Cell apoptosis rates in the shRNA-1 and shRNA-2 groups were higher than that in the CDA mimic group. **a** Cell apoptosis was detected by flow cytometry. The left lower quadrant of flow cytometry scatter plot represented healthy living cells (FITC−/PI−), the left upper quadrant represented the cells (FITC−/PI+) with mechanical damage, the right lower quadrant represented the cells (FITC+/PI−) in early apoptosis, and the right upper quadrant represented the cells (FITC+/PI+) in necrotic and late apoptotic stages. The apoptosis rate = percentage of cells in early apoptosis + percentage of cells in advanced apoptosis; **b** histogram analysis for the rates of early apoptosis (FITC+/PI−) and late apoptosis (FITC+/PI+) in each group; the data were expressed as mean ± standard deviation, n = 3, and One-Way ANOVA was used to analyzed the data. *Compared to the blank group, *p *< 0.05. *CDA* cytidine deaminase, *FITC* fluorescein isothiocyanate, *PI* propidium iodide

Cell apoptosis was detected by flow cytometry. As shown in Fig. 6 and after 48 h of transfection, the cell apoptosis in the blank, shRNA-con, shRNA-1, shRNA-2 and group of over-expressed CDA was (3.71 ± 0.45)%, (3.77 ± 0.23)%, (9.69 ± 0.68)%, (9.76 ± 0.45)% and (1.36 ± 0.13)%, respectively. When compared with the blank group, the apoptotic rate of K562 cells decreased significantly upon CDA overexpression and increased significantly after CDA gene silencing (all *p* < 0.05). Therefore, CDA gene silencing promoted cell apoptosis.

### CDA gene silencing down-regulated BCL-2 and up-regulated p21, Bax, cleaved caspase-3/total caspase-3 and cleaved PARP/total PARP

**Fig. 7:**
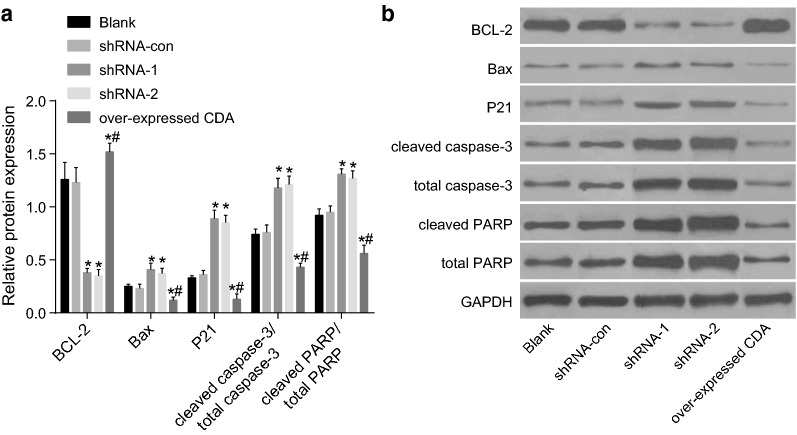
CDA gene silencing down-regulated BCL-2 and up-regulated p21, Bax, cleaved caspase-3/total caspase-3 and cleaved PARP/total PARP. **a** Protein bands of BCL-2, Bax, p21, cleaved caspase-3, total caspase-3, cleaved PARP, total PARP and GAPDH in each group; **b** protein expression of BCL-2, Bax, P21, cleaved caspase-3, total caspase-3, cleaved PARP, total PARP and GAPDH in each group; the data were expressed as mean ± standard deviation, n = 3, and One-Way ANOVA was used to analyzed the data. *Compared with the blank group, *p *< 0.05. *CDA* cytidine deaminase, *BCL-2* B-cell lymphoma/leukemia-2, *Bax* BCL-2-associated X, *PARP* poly (ADP-ribose) polymeras, *GAPDH* glyceraldehyde-3-phosphate dehydrogenase

Western blot analysis was used to detect the protein expression of genes related to cell proliferation, apoptosis and cell cycle. As shown in Fig. 7 (after 48 h of transfection) and compared with the blank group, the expression of relevant genes in the shRNA-con group showed no obvious difference. In addition, the BCL-2 expression in the shRNA-1 and shRNA-2 groups decreased obviously (all *p* < 0.05), while the expression of p21, Bax, cleaved caspase-3/total caspase-3 and cleaved PARP/total PARP increased obviously (all *p* < 0.05). ShRNA-1 and shRNA-2 groups showed no obvious difference in terms of the expression of above genes (*p* > 0.05). BCL-2 expression obviously increased in the CDA mimic group, while the expression of P21, Bax, cleaved caspase-3/total caspase-3 and cleaved PARP/total PARP significantly decreased (all *p* < 0.05). Therefore, CDA gene silencing decreased the protein expression of BCL-2 and increased the protein expression of P21, Bax, cleaved caspase-3/total caspase-3, and cleaved PARP/total PARP.

### Underlying mechanism of CDA in K562 cell proliferation, apoptosis and cell cycle

**Fig. 8:**
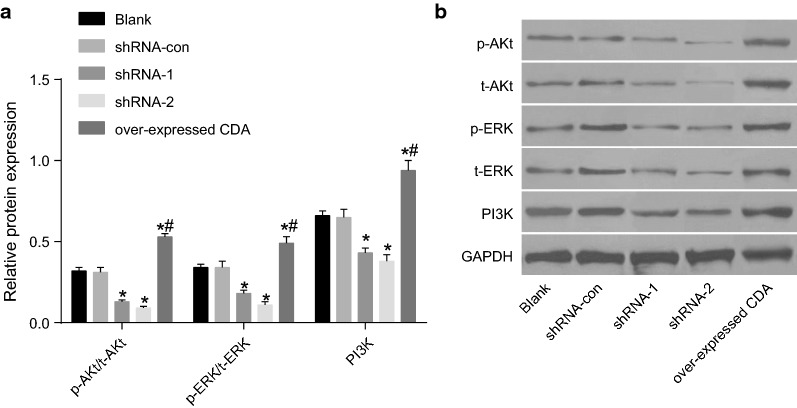
Mechanism of CDA in the proliferation, apoptosis and cell cycle of K562 cells. **a** Protein bands of PI3K, p-AKT/t-AKT, p-ERK1/2/t-ERK1/2 and GAPDH in each group; **b** protein expression of PI3K, p-AKT/t-AKT, p-ERK1/2/t-ERK1/2; the data were expressed as mean ± standard deviation, n = 3, and One-Way ANOVA was used to analyzed the data. *Compared with the blank group, *p *< 0.05. *PI3K* phosphatidylinositol-3 kinase, *ERK* extracellular signal-regulated kinase, *CDA* cytidine deaminase, *GAPDH* glyceraldehyde-3-phosphate dehydrogenase

Western blot analysis was used to detect the protein expression of genes related to the PI3K/Akt signaling pathway and the MAPK/ERK signaling pathway. As shown in Fig. 8 (after 48 h of transfection) and compared with the blank group, the shRNA-con group showed no obvious difference in the protein expression of relevant genes. In the shRNA-1 and shRNA-2 groups, the expression of PI3K, p-AKT/t-AKT, and p-ERK1/2/t-ERK1/2 obviously decreased (all *p* < 0.05), while the shRNA-1 and shRNA-2 groups showed no obvious difference in terms of the expression of above genes (*p* > 0.05). In the group of over-expressed CDA, the expression of PI3K, p-AKT/t-AKT, p-ERK1/2/t-ERK1/2 obviously increased (all *p* < 0.05). Thereby, CDA gene silencing may reduce the expression of PI3K, p-AKT/t-AKT, and p-ERK1/2/t-ERK1/2.

### CDA gene silencing inhibited tumor growth

**Fig. 9:**
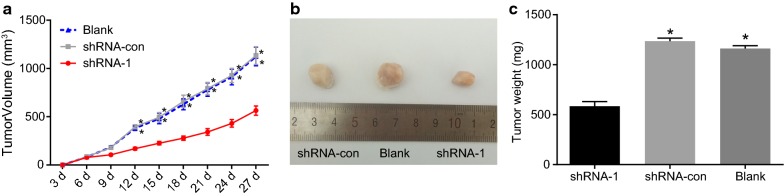
CDA gene silencing inhibited tumor growth. For nude mice inoculated with K562 cells, the tumor growth rate in the shRNA-1 group was slower than that in the blank and shRNA-con groups. **a** Tumor volume in each group; **b** tumor formation in each group; **c** tumor weight in each group; the data were expressed as mean ± standard deviation, n = 3, and One-Way ANOVA was used to analyzed the data. *Compared with the shRNA-1 group, *p *< 0.05; CDA, cytidine deaminase

In order to further detect the effect of CDA on the growth and development of glioma cells, K562 cells treated by different plasmids were inoculated into nude mice. As shown in Fig. 9, different groups of nude mice were all able to form a xenograft tumor. The tumor growth in nude mice inoculated with blank and shRNA-con cells was faster than that in mice inoculated with shRNA-1 cells (all *p* < 0.05). In addition, the weight and volume of tumor were calculated for each group. The volume and weight of tumor in the shRNA-1 group were significantly less than those in the blank and shRNA-con groups (*p* < 0.05). Therefore, CDA gene silencing inhibited tumor growth.

## Discussion

It seems that CML mainly affects young people from Asia [17]. A previous study revealed that high expression of CDA was responsible for cytidine resistance in cancer cells [18]. Therefore, the aim of our study was to determine the effects of CDA gene silencing on the apoptosis and proliferation of K562 cells. Besides, our study confirmed that down-regulated CDA gene expression could inhibit cell proliferation and induce apoptosis.

In our study, the mRNA expression and enzymatic activity of CDA in CML and AL patients remarkably increased compared with those in the donors of hematopoietic stem cell transplantation. CDA is a widely used chemotherapeutic agent in the treatment of certain leukemia and has high expression in cancer cells, which is regarded as a prominent enzyme in the catabolism of cytosine nucleoside analogues [19, 20]. In addition, CDA was reported to be the prominent enzyme in the metabolism of gemcitabine, a pyrimidine analog widely used in the chemotherapy of tumors such as biliary tract cancer. Therefore, the cellular activity of CDA is crucial for understanding gemcitabine-induced toxicity [10, 11]. Whether in vitro or in vivo, CDA could play a major role to regulate the susceptibility to antitumor dCyd analogs and hence affect the antitumor activity of 2′-C-cyano-2′-deoxy-1-beta-d-arabinofuranosylcytosine (CNDAC) [21]. One former study found that, in many cancers including myeloid leukemia, the CDA expression in male cancer patients was remarkably higher than that in females [22]. Besides, when compared with patients of complete remission, CDA expression was higher in previously untreated and relapsed AL patients [23]. Additionally, in AML patients, lower CDA levels predicted a longer disease-free survival [24]. Carpi et al. demonstrated that the average value of CDA activity was 0.051 ± 0.024 mU/mg, which also followed a normal distribution in healthy subjects [25]. For this reason, we speculated that CDA could become a promising biomarker and therapeutic target for the treatment of leukemia.

In order to study the effect of CDA gene on the biological property of K562 cells, we carried out CDA gene silencing in K562 cells. Upon CDA gene silencing, the cell proliferation was inhibited and the cells were arrested in S and G2/M phases, accompanied by an increased apoptotic rate. CDA treatment can cause the death of Jurkat human T leukemia cells by inducing apoptosis in a time- and dose-dependent fashion [19]. A previous study found that miR-484 could inhibit CDA and suppress the proliferation of breast cancer cells [26]. Furthermore, when compared with the blank group, the shRNA-1 and shRNA-2 groups were associated with down-regulated expression of PI3K, p-AKT/t-AKT, p-ERK1/2/t-ERK1/2 and Bcl-2, and up-regulated expression of p21, Bax, cleaved caspase-3/total caspase-3 and cleaved PARP/total PARP. It implicated that CDA gene silencing could involve in the ability of K562 cell proliferation and apoptosis by regulating the PI3K/Akt signaling pathway and MAPK/ERK signaling pathway PARPs were found to promote the transformation of ADP-ribose and play critical roles in several cellular processes, such as transcription, replication, recombination, and DNA repair [27]. A recent study found that CDA deficiency could impair sister chromatid disjunction by decreasing PARP-1 activity [28]. ERK is important for transducing extracellular signals to cell nuclei, and hence acts as a main downstream transducer of Ras. In addition, the invasiveness of tumor cells can be altered by nuclear localization of phosphorylated or activated ERK [29]. CDA treatment has been reported to increase the phosphorylation of ERK1/2 in Jurkat T leukemia cells [19]. It was reported that the chronic phase of CML and the blast crisis of CML were accompanied by AKT overexpression [30]. It has also been reported that the reduction in AKT expression enhanced gemcitabine-induced apoptosis and its anti-tumor activity in pancreatic carcinoma [31]. PI3Ks are lipid kinases that can regulate various cellular processes, such as proliferation, adhesion and survival [32]. PI3Ks are frequently upregulated in cancer [33], suggesting that the down-regulated expression of PI3K upon CDA gene silencing may be helpful for the treatment of CML. Our study agrees well with the above, that CDA gene silencing mediated the PI3K/Akt signaling pathway and MAPK/ERK signaling pathway, resulting in under controlled proliferation of K562 cells. The mediation of PI3K/Akt signaling pathway and MAPK/ERK signaling pathway on apoptotic factors such as Bax and Bcl-2 have been illustrated in lots of studies. The Bcl-2 family proteins are important regulators and their main acting site is on the outer mitochondrial membrane [34]. As an anti-apoptotic protein, the high-expression of Bcl-2 could impede apoptosis in cancer cells [35]. A previous study found that the expression of pro-apoptotic factors, Bax and Caspase3, was up-regulated in chemotherapy, while the expression of anti-apoptotic factor Bcl-2 was markedly down-regulated [36]. As a cyclin-dependent kinase inhibitor, p21 can stop cellular proliferation and block cell cycle progression [37]. As a member of the Bcl-2 family, Bax is a pro-apoptotic factor translocated from the cytosol to the mitochondria during programmed cell death [38]. As a result, we observed activation of anti-apoptotic factor Bcl-2, together with suppression of pro-apoptotic factor Bax. In related to silenced CDA, K562 cell proliferation is restricted, and apoptosis is accelerated.

## Conclusions

In conclusion, our study demonstrated that CDA was highly expressed in CML cells, and CDA gene silencing could inhibit the proliferation of CML cells and promote their apoptosis. These processes might be closely associated with the development of malignant tumors. Thus, CDA could become a new target for the treatment of CML. However, we have not studied other cell lines due to limited time and resources, and the specific mechanism underlying the function of CDA in CML remains elusive.

## Abbreviations

CML: chronic myeloid leukemia
RT-qPCR: reverse transcription-quantitative polymerase chain reaction
CDA: cytidine deaminase
AL: acute leukemia
DECP: diethylpyrocarbonate
CCPM: corrected counts per minute
